# Biochemical and computational analyses of two phenotypically related GALT mutations (S222N and S135L) that lead to atypical galactosemia

**DOI:** 10.1016/j.dib.2015.01.001

**Published:** 2015-02-07

**Authors:** Benjamin Cocanougher, Umut Aypar, Amber McDonald, Linda Hasadsri, Michael J. Bennett, W. Edward Highsmith, Kristin D׳Aco

**Affiliations:** a University of Rochester School of Medicine and Dentistry, Department of Pediatrics, Division of Genetics, Rochester, NY, USA; b Molecular Genetics Laboratory, Department of Laboratory Medicine and Pathology, Mayo Clinic, Rochester, MN, USA; c Metabolic Disease Laboratory, Children׳s Hospital of Philadelphia, USA; d Department of Pathology and Laboratory Medicine, Perelman School of Medicine at the University of Pennsylvania, Philadelphia, PA, USA

## Abstract

Galactosemia is a metabolic disorder caused by mutations in the *GALT* gene [1,2]. We encountered a patient heterozygous for a known pathogenic H132Q mutation and a novel S222N variant of unknown significance [3]. Reminiscent of patients with the S135L mutation, our patient had loss of GALT enzyme activity in erythrocytes but a very mild clinical phenotype [3–8]. We performed splicing experiments and computational structural analyses to investigate the role of the novel S222N variant. Alamut software data predicted loss of splicing enhancers for the S222N and S135L mutations [9,10]. A cDNA library was generated from our patient׳s RNA to investigate for splicing errors, but no change in transcript length was seen [3]. *In silico* structural analysis was performed to investigate enzyme stability and attempt to understand the mechanism of the atypical galactosemia phenotype. Stability results are publicly available in the GALT Protein Database 2.0 [11–14]. Animations were created to give the reader a dynamic view of the enzyme structure and mutation locations. Protein database files and python scripts are included for further investigation.

**Specifications table**

**Table:** 

Subject area	Medicine

More specific subject area	Medical genetics, galactosemia
Type of data	*Graph, protein structure animation, pdb files, python scripts*
How data was acquired	*In silico* structural analyses performed with Chimera (UCSF) and modeller programs. Splicing studies performed with Alamut Splicing Predictor (Cold Spring Harbor Laboratories). RNA studies performed on whole blood sample from patient.
Data format	Analyzed molecular data, Analyzed computational data, and raw.pdb files for structural analysis
Experimental factors	Computational analyses were performed using Alamut software, Modeller, and UCSF Chimera.
Experimental features	Splicing enhancer studies performed using Alamut software. Animations and structural analysis performed with Modeller and UCSF Chimera software.
Data source location	Not applicable
Data accessibility	Structural data available in GALT protein database (GALT Protein Database 2.0). Biochemical data available in article.

**Value of the data**

• Heterozygosity for novel S222N GALT mutation leads to atypical galactosemia similar to known S135L cases.

• **Splicing enhancers are predicted to be lost for both S222N and S135L mutations.**

• *In silico* analyses predict stability/instability of dimers present in our patient and allow for visualization of mutation types.

## 1 Data, experimental design, materials and methods

### 1.1 Case report

Our data is based on a single case of atypical galactosemia caused by compound heterozygosity for a known pathogenic H132Q mutation and a novel S222N variant of unknown significance [3]. Briefly, our patient had a positive newborn screen for galactosemia with subsequent biochemical confirmation of absent GALT enzyme activity, was treated with a galactose-restricted diet until the age of 3 with no symptoms, and was later lost to follow-up. The patient presented to our clinic at age 17 with no stigmata of galactosemia (apart from mild anxiety) to determine his disease status [15]. Testing revealed nearly complete absence of GALT enzyme activity in erythrocytes without any increase in the clinical biomarkers of galactosemia, galactose-1-phosphate and galactitol [16,17]. Sequencing revealed compound heterozygosity for H132Q and S222N mutations.

Given the ability of our patient to oxidize galactose in tissues other than red blood cells, as evidenced by galactose-1-phosphate and galactitol levels in the normal range, we suspected a splicing defect. Differential splicing seemed a reasonable explanation for differential tissue activity of the GALT enzyme.

### 1.2 Splicing experiments

**Fig. 1:**
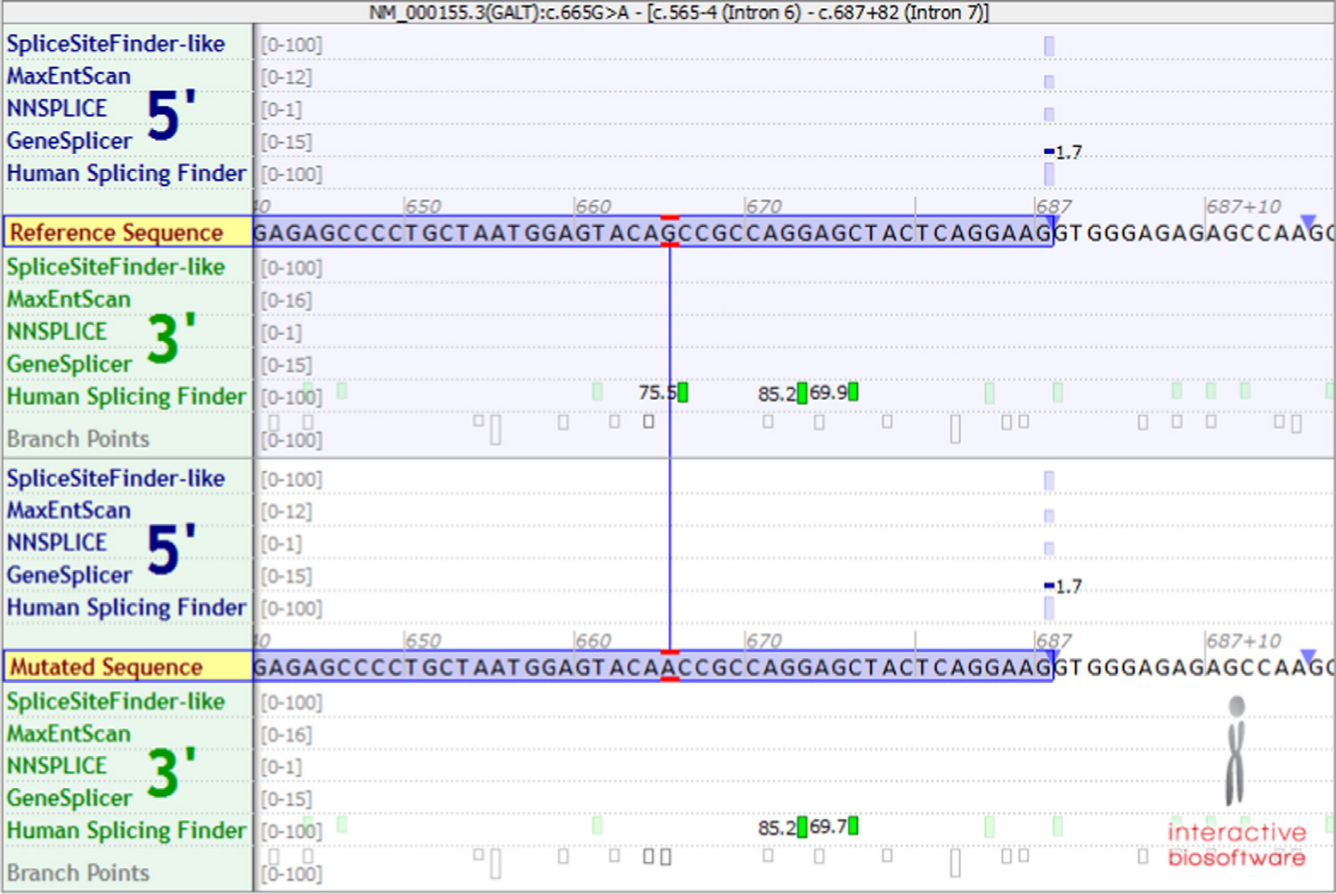
Screenshot from the Alamut (Cold Spring Harbor Laboratories) Splicing Effects window around the GALT c665G>A variant [9,10]. The top box represents the wild type sequence, with a G at position c.665, and the bottom panel representing the mutant sequences, with an A at c.665. In both panels the nucleotides boxed in lavender represent the 3′ end of exon 7. The mutant sequence is not predicted to create or destroy a predicted splice site.

We first examined the S222N mutation for changes in splice sites due to genomic c.665G>A. Fig. 1 is a screenshot of the Alamut software program showing that this mutation does not create or destroy a predicted splice site [9,10].

**Fig. 2:**
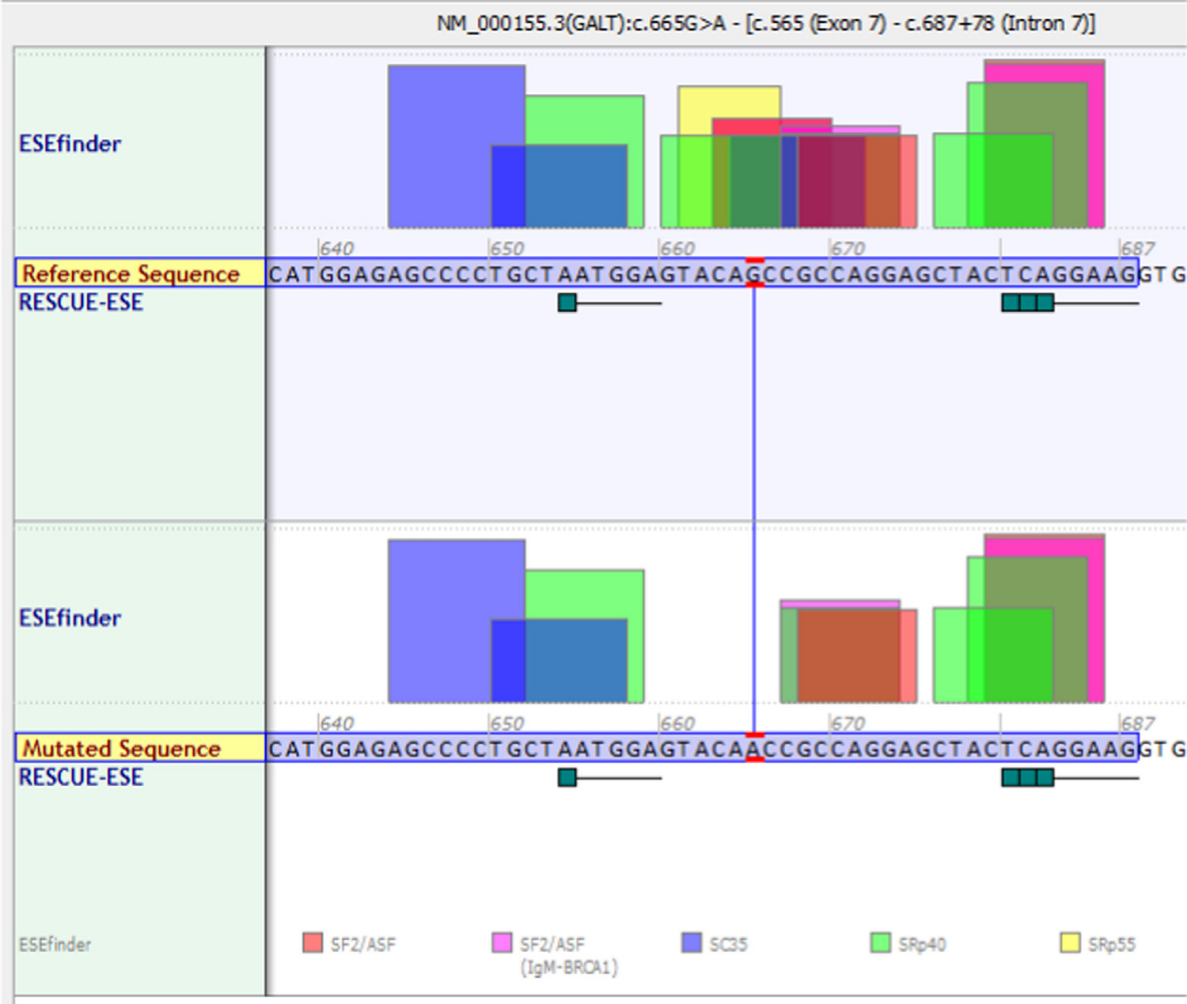
Screenshot from the Alamut (Cold Spring Harbor Laboratories) Splicing Predictor/ESE Predictions window around the GALT c665G>A variant [9,10]. The top box represents the wild type sequence, with a G at position c.665, and the bottom panel representing the mutant sequences, with an A at c.665. In both panels the nucleotides boxed in lavender represent the 3′ end of exon 7. The mutant sequence is predicted to lose binding sites for SRp40 (green box), SRp55 (yellow box), and SF2/ASF (pink/red box).

**Fig. 3:**
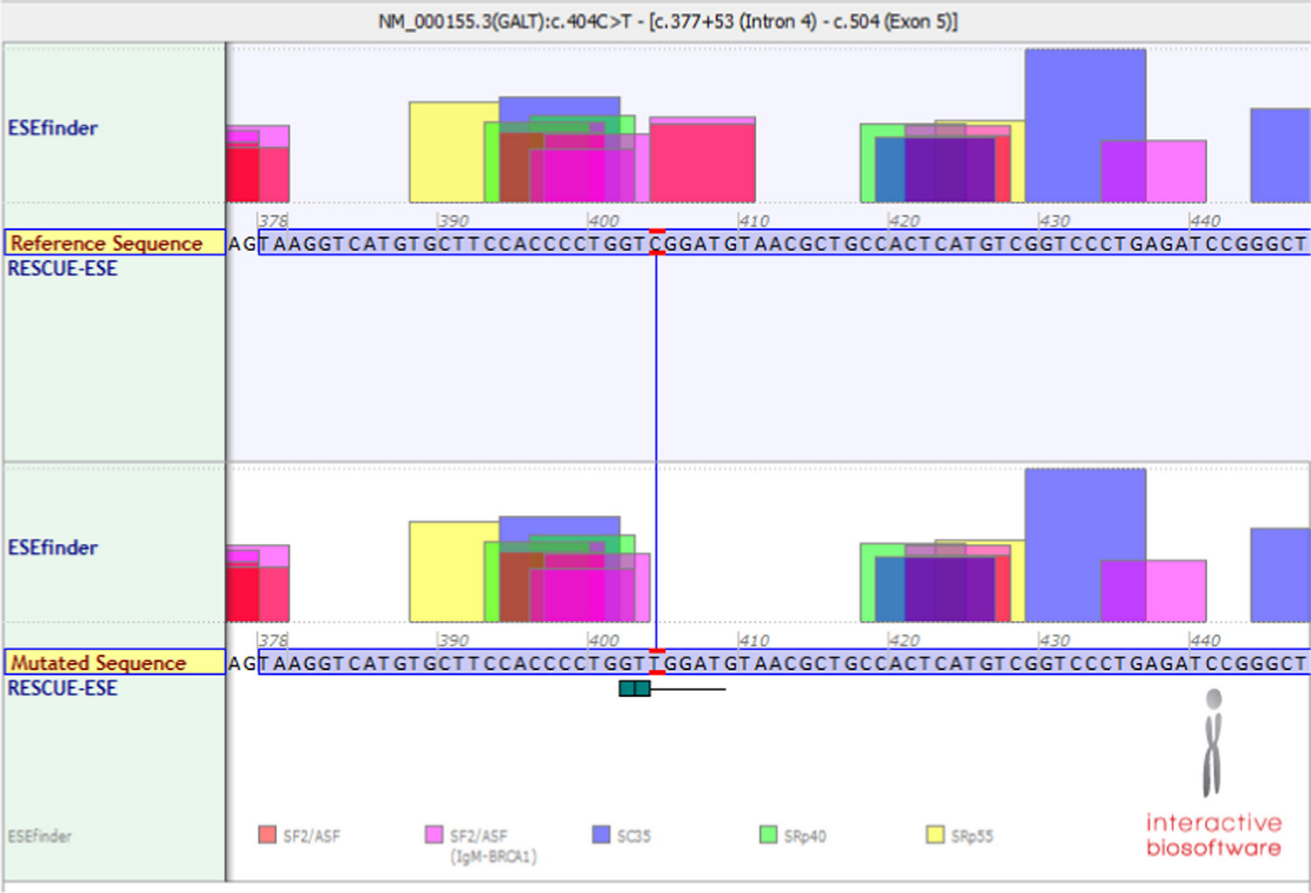
Screenshot from the Alamut (Cold Spring Harbor Laboratories) Splicing Predictor/ESE Predictions window around the GALT c.404 C>T variant responsible for the S135L mutation [8,9]. The top box represents the wild type sequence, with a G at position c.665, and the bottom panel representing the mutant sequences, with an A at c.665. In both panels the nucleotides boxed in lavender represent the 3′ end of exon 7. The mutant sequence is predicted to lose binding sites for SF2/ASF (pink/red box).

Though this mutation does not directly lead to changes in a splice site, further investigation revealed a predicted loss of a cluster of exonic splicing enhancer (ESE) sites due to the c.665G>A mutation (Fig. 2). This could potentially explain the loss of activity only in erythrocytes if these enhancers were specific to red blood cells and unused in other tissues. Interestingly, the c.404C>T mutation, which leads to the S135L protein, also results in a predicted loss of ESE clusters (Fig. 3).

In order to further investigate this hypothesis, RNA was isolated from the whole blood sample, a cDNA library was created, and PCR was used to amplify the entire gene in several overlapping pieces. No shift in cDNA size was seen, which would be expected in the case of interrupted or aberrant splicing (Supplementary Fig. 1) [3]. The cDNA was sequenced to look for splicing defects that may not be apparent on gel electrophoresis, but there were no changes in exon structure from the reference or the father׳s cDNA sequence (Supplementary Fig. 2) [3]. The only base pair change identified was the c.665G>A variant itself.

### 1.3 Structure analysis

Protein modeling was performed to evaluate the stability of the three types of dimers produced by our patient (H132Q homodimer, H132Q/S222N heterodimer, and S222N homodimer) and the phenotypically similar S135L homodimer (Supplementary Files 1–4). We submitted our mutation to the GALT protein database run by Dr. Anna Marabotti and the Laboratory of Bioinformatics at the Institute of Food Science. The models were mutated using the “mutate model” function in Modeller. Data on stability of each of the various GALT dimers are are accessible in the GALT Protein Database 2.0 [11,12].

**Fig. 4:**
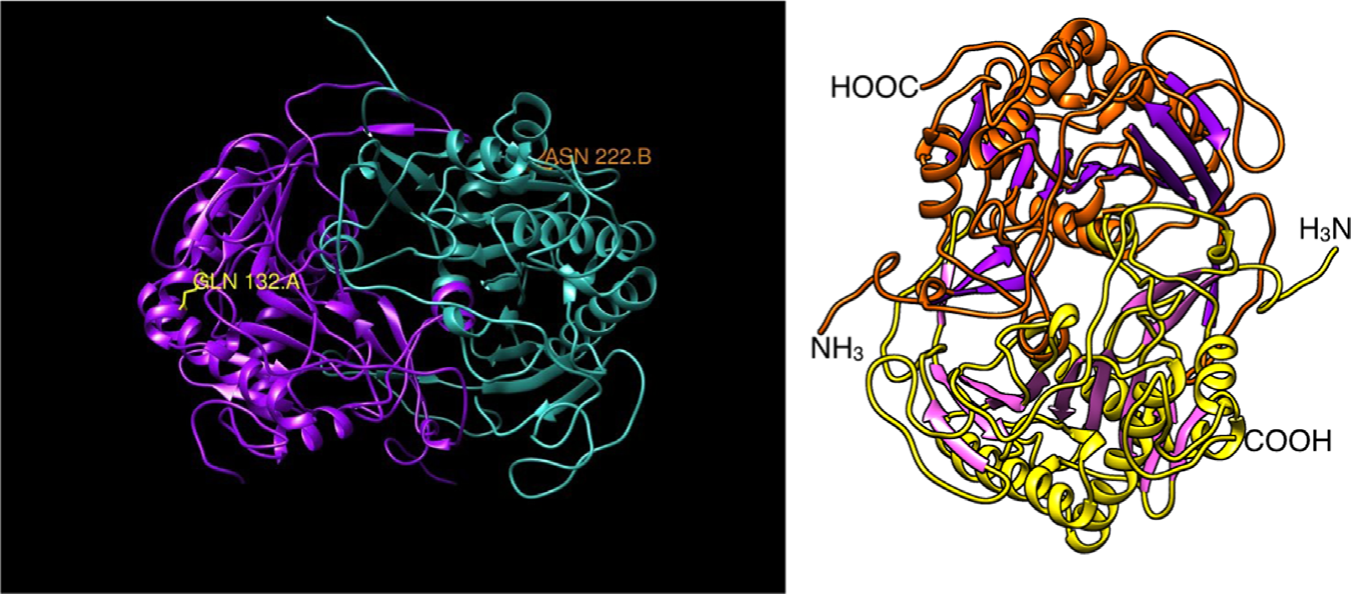
Video stills of two movies generated to examine the structure of GALT enzyme variants. Supplementary Movie 1 examines the location and neighboring residues for the H132Q/S222N heterodimer. Supplementary Movie 2 examines the location of the S222N and S135L mutation residues.

Using the UCSF Chimera software, we investigated the residues involved in our four dimeric structures. As evidenced in the included animations, the mutations in the H132Q and S222N heterodimer are too far from one another in the structure to interact directly (Supplementary Movie 1) (Fig. 4).

A separate analysis was performed to visualize the S222N and S135L residue locations. The two are located on separate ends of the dimer, but both appear to be peripherally accessible for phosphorylation in the non-mutated state (Supplementary Movie 2). This animation was colored to match the original publication of the GALT enzyme structure [18]. Python scripts created to generate both of the animations above have been included (Supplementary Files 5,6). They can be opened and modified using the UCSF Chimera software [14].

## Supplementary materials

Supplementary data: Supplementary Movie 1: Animation of the H132Q/S222N heterodimer. This animation was created using the UCSF Chimera software Animation tool [14]. It highlights the location and local molecular environment of the two mutations seen in our patient. The protein database file for this GALT structure (Supplementary File 2) and python script used to create this animation (Supplementary File 5) are included. Supplementary Movie 2: Animation highlighting the location of the S222N and S135L mutations. This animation was created using the UCSF Chimera software Animation tool [14]. It highlights the location and local molecular environment of the two mutations known that result in loss of GALT enzyme activity in erythrocytes without loss of whole body galactose oxidation. The protein database file for this GALT structure (Supplementary File 3) and python script used to create this animation (Supplementary File 6) are included. Supplementary Fig. 1: RT-PCR electrophoresis shows no shift in *GALT* cDNA size between father, patient, and control (HeLa) cells. Supplementary Fig. 2: cDNA sequencing shows exons 6, 7, and 8 are intact with no exon skipping event. The S222N variant of uncertain significant (VUS) was detected. Supplementary File 1: GALT H132Q homodimer.pdb Raw data used to generate GALT protein model. This file was submitted by Dr. Anna Marabotti and created by using the “mutate model” function of the Modeller program [11–13]. This model can be opened with any modeling software that accepts protein database files [14]. Supplementary File 2: GALT H132Q S222N Heterodimer.pdb Raw data used to generate GALT protein model. This file was submitted by Dr. Anna Marabotti and created by using the “mutate model” function of the Modeller program [11–13]. This model can be opened with any modeling software that accepts protein database files [14]. Supplementary File 3: GALT S135L homodimer.pdb Raw data used to generate GALT protein model. This file was submitted by Dr. Anna Marabotti and created by using the “mutate model” function of the Modeller program [11–13]. This model can be opened with any modeling software that accepts protein database files [14]. Supplementary File 4: GALT S222N homodimer.pdb Raw data used to generate GALT protein model. This file was submitted by Dr. Anna Marabotti and created by using the “mutate model” function of the Modeller program [11–13]. This model can be opened with any modeling software that accepts protein database files [14]. Supplementary File 5: Animation 1.py This python script can be opened with the UCSF Chimera software [14]. It includes correctly colored models from the animations with all of the animation views available with the “Animation” tool. Additional analyses of the structure can be performed with this code. Supplementary File 6: Animation 2.py This python script can be opened with the UCSF Chimera software [14]. It includes correctly colored models from the animations with all of the animation views available with the “Animation” tool. Additional analyses of the structure can be performed with this code.
