# Supplementary figures and images for: Biochemical and computational analyses of two phenotypically related GALT mutations (S222N and S135L) that lead to atypical galactosemia
Source: Data Brief. 2015 Feb 7;3:34–9. doi: 10.1016/j.dib.2015.01.001 (PMC4509990; doi:10.1016/j.dib.2015.01.001)

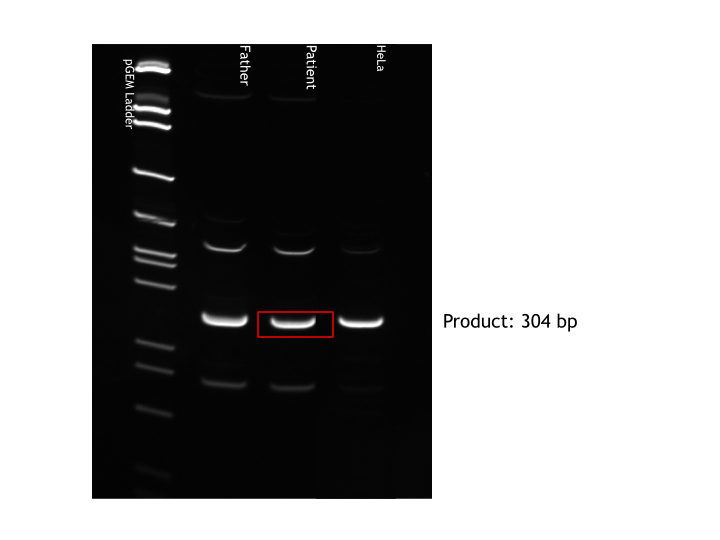

Supplement: Supplementary file 1 — Supplementary data: Supplementary Movie 1: Animation of the H132Q/S222N heterodimer. This animation was created using the UCSF Chimera software Animation tool [14]. It highlights the location and local molecular environment of the two mutations seen in our patient. The protein database file for this GALT structure (Supplementary File 2) and python script used to create this animation (Supplementary File 5) are included. Supplementary Movie 2: Animation highlighting the location of the S222N and S135L mutations. This animation was created using the UCSF Chimera software Animation tool [14]. It highlights the location and local molecular environment of the two mutations known that result in loss of GALT enzyme activity in erythrocytes without loss of whole body galactose oxidation. The protein database file for this GALT structure (Supplementary File 3) and python script used to create this animation (Supplementary File 6) are included. Supplementary Fig. 1: RT-PCR electrophoresis shows no shift in GALT cDNA size between father, patient, and control (HeLa) cells. Supplementary Fig. 2: cDNA sequencing shows exons 6, 7, and 8 are intact with no exon skipping event. The S222N variant of uncertain significant (VUS) was detected. Supplementary File 1: GALT H132Q homodimer.pdb Raw data used to generate GALT protein model. This file was submitted by Dr. Anna Marabotti and created by using the “mutate model” function of the Modeller program [11–13]. This model can be opened with any modeling software that accepts protein database files [14]. Supplementary File 2: GALT H132Q S222N Heterodimer.pdb Raw data used to generate GALT protein model. This file was submitted by Dr. Anna Marabotti and created by using the “mutate model” function of the Modeller program [11–13]. This model can be opened with any modeling software that accepts protein database files [14]. Supplementary File 3: GALT S135L homodimer.pdb Raw data used to generate GALT protein model. This file was submitted by [file mmc1.zip › BTC Supplementary files/Supplementary Figure 1.jpg]

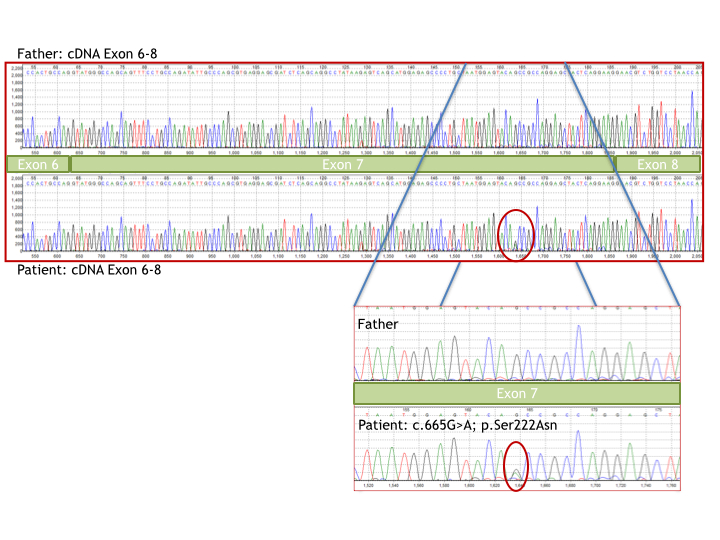

Supplement: Supplementary file 1 — Supplementary data: Supplementary Movie 1: Animation of the H132Q/S222N heterodimer. This animation was created using the UCSF Chimera software Animation tool [14]. It highlights the location and local molecular environment of the two mutations seen in our patient. The protein database file for this GALT structure (Supplementary File 2) and python script used to create this animation (Supplementary File 5) are included. Supplementary Movie 2: Animation highlighting the location of the S222N and S135L mutations. This animation was created using the UCSF Chimera software Animation tool [14]. It highlights the location and local molecular environment of the two mutations known that result in loss of GALT enzyme activity in erythrocytes without loss of whole body galactose oxidation. The protein database file for this GALT structure (Supplementary File 3) and python script used to create this animation (Supplementary File 6) are included. Supplementary Fig. 1: RT-PCR electrophoresis shows no shift in GALT cDNA size between father, patient, and control (HeLa) cells. Supplementary Fig. 2: cDNA sequencing shows exons 6, 7, and 8 are intact with no exon skipping event. The S222N variant of uncertain significant (VUS) was detected. Supplementary File 1: GALT H132Q homodimer.pdb Raw data used to generate GALT protein model. This file was submitted by Dr. Anna Marabotti and created by using the “mutate model” function of the Modeller program [11–13]. This model can be opened with any modeling software that accepts protein database files [14]. Supplementary File 2: GALT H132Q S222N Heterodimer.pdb Raw data used to generate GALT protein model. This file was submitted by Dr. Anna Marabotti and created by using the “mutate model” function of the Modeller program [11–13]. This model can be opened with any modeling software that accepts protein database files [14]. Supplementary File 3: GALT S135L homodimer.pdb Raw data used to generate GALT protein model. This file was submitted by [file mmc1.zip › BTC Supplementary files/Supplementary Figure 2.jpg]
